# Application of Meat-Derived Lactic Acid Bacteria Strains to Improve the Quality of Organic Fermented Beef Sausages

**DOI:** 10.3390/molecules31111948

**Published:** 2026-06-04

**Authors:** Anna Łepecka, Aleksandra Szydłowska, Katarzyna Marciniak-Lukasiak, Anna Okoń, Olga Świder, Sylwia Onacik-Gür, Beata Łaszkiewicz, Urszula Siekierko, Dorota Grzeszczak, Piotr Szymański

**Affiliations:** 1 Department of Meat and Fat Technology, Institute of Agricultural and Food Biotechnology—State Research Institute, 02-532 Warsaw, Poland; anna.okon@ibprs.pl (A.O.); sylwia.onacik-gur@ibprs.pl (S.O.-G.); beata.laszkiewicz@ibprs.pl (B.Ł.); urszula.siekierko@ibprs.pl (U.S.); dorota.grzeszczak@ibprs.pl (D.G.); piotr.szymanski@ibprs.pl (P.S.); 2 Department of Food Gastronomy and Food Hygiene, Institute of Human Nutrition Sciences, Warsaw University of Life Sciences, 02-776 Warsaw, Poland; aleksandra_szydlowska@sggw.edu.pl; 3 Department of Food Technology and Assessment, Institute of Food Sciences, Warsaw University of Life Sciences, 02-776 Warsaw, Poland; katarzyna_marciniak-lukasiak@sggw.edu.pl; 4 Department of Food Safety and Chemical Analysis, Institute of Agricultural and Food Biotechnology—State Research Institute, 02-532 Warsaw, Poland; olga.swider@ibprs.pl

**Keywords:** fermentation, maturing, meat products, cold cuts, *Lactiplantibacillus plantarum*, *Lactiplantibacillus pentosus*, starter culture, autochthonous

## Abstract

This study aimed to assess the potential of meat-derived lactic acid bacteria (LAB) strains on the technological, microbiological, and physicochemical quality of beef sausages. Four fermented sausage treatments were prepared: (C) was produced without cultures; (S2A) was produced with *Lactiplantibacillus plantarum* S2A; (S4B) was produced with *Lactiplantibacillus pentosus* S4B; and (OP4) was produced with *Lactiplantibacillus plantarum* OP4. All tested treatments were characterized by high total aerobic mesophilic and LAB counts (7.48–8.16 and 7.68–8.20 log CFU/g, respectively). Overall, the sausages were characterized by insufficient microbiological quality, with a relatively high number of coagulase-positive staphylococci (3.35–4.26 log CFU/g). The sausages differed significantly in terms of color (*p* < 0.05). The C and S4B treatments were shown to be more red and yellow than S2A and OP4. Differences were observed in the texture assessment of the sausages, and the least hard were those of the OP4 treatment (288.01 N). Analysis of physicochemical parameters revealed no significant differences in water activity (*p* > 0.05). The starter culture treatments were characterized by a higher pH (5.69–5.82; *p* < 0.05) than the C treatment (5.42). Oxidation–reduction potential was significantly higher in the control sample (469.87 mV; *p* < 0.05). The highest peroxide value and TBARS (thiobarbituric acid reactive substances) values were recorded in S2A (1.98 meq O_2_ kg^−1^ of fat and 1.784 mg MDA kg^−1^, respectively), while the lowest found in S4B and OP4 (1.65 meq O_2_ kg^−1^ of fat and 1.340 mg MDA kg^−1^, respectively). Fatty acid profile analysis revealed that the use of LAB influenced the proportion of individual lipid fractions in fermented beef sausages. Free amino acid analysis revealed a significant effect of the LAB starter cultures used on the intensity of proteolytic transformations in sausages. The results indicate that indigenous strains of LAB can be effectively used as starter cultures in the production of fermented beef sausages. Their use contributes to improving the product’s physicochemical and textural properties and may also increase its oxidative stability and nutritional value.

## 1. Introduction

Fermented beef sausages constitute an important group of meat products whose shelf life, microbiological safety, and sensory characteristics are shaped by the process of lactic acid fermentation and maturation [1].

The production of fermented beef products is more demanding than that of pork due to the higher protein content, lower intramuscular fat content, and different fatty acid composition [2,3]. These characteristics influence the product’s texture, oxidative stability, and the development of desired sensory characteristics [4,5]. Additionally, beef contains small amounts of residual sugars and is characterized by a higher pH, which may hinder the growth of LAB, slow down acidification, and limit the product’s microbiological stability [6,7,8].

Organic production is based on the principles of sustainable development, environmental protection, and animal welfare [9]. In the case of meat production, this primarily means limiting the use of nitrites (mainly in the form of sodium nitrite (E250)) in meat processing. The role of preservatives is primarily to inhibit *Clostridium botulinum* but also to stabilize oxidative processes, create the appropriate red color, and shape the taste and aroma of meat products [10]. The use of nitrites raises health concerns, and for this reason, their amount in food products is strictly regulated by law [11]. Therefore, producers are seeking alternative preservation methods, and one method is the use of LAB cultures [12].

Microorganisms play an important role during the fermentation and maturation of meat products, dominated by LAB, coagulase-negative staphylococci (CNS), yeasts, molds, and other associated bacteria [13,14]. Their role is to stabilize the product and ensure microbiological safety through biochemical acidification of the environment, the production of bacteriocins and hydrogen peroxide, and environmental competition, thus extending the shelf life [15]. Furthermore, microbes shape the sensory characteristics of the product and stabilize color as a result of the action of oxidoreductive enzymes and texture profiling [8,16]. In turn, according to research by Cheng et al. [17], *Staphylococcus* spp. and *Macrococcus* spp. are crucial in shaping the overall quality of beef and contribute to its exceptional palatability and aroma. During meat maturation, intensive proteolysis and lipolysis processes occur, catalyzed by both endogenous enzymes and enzymes produced by microorganisms. The breakdown of proteins and fats leads to the formation of more easily digestible compounds, while also contributing to the characteristic aroma, delicate texture, and juiciness of the product, which significantly determines its attractiveness to consumers [17].

Meat starter cultures are selected microorganisms whose function is to control biological processes occurring during meat processing [13]. In artisanal practices, the lack of control over environmental conditions and the risk of contamination can result in an inconsistent quality of products subjected to spontaneous fermentation [14]. In the case of meat starters, these include primarily LAB with GRAS (Generally Recognized As Safe) status and microorganisms that support nitrite reduction and color development. The use of starter cultures ensures greater process repeatability and predictability [13]. Despite the widespread use of LAB in meat processing, relatively little research has focused on their use in fermented beef sausages. This study aimed to assess the potential of meat-derived LAB strains on the technological, microbiological, and physicochemical quality of fermented beef sausages. It was hypothesized that indigenous strains of LAB, isolated directly from fermented meat products, would be better adapted to the specific conditions of the meat environment than cultures not originating from this environment. Their use could promote a more controlled fermentation process by more effectively acidifying the environment, limiting the growth of undesirable microflora, and influencing the proteolytic and lipid transformations occurring during product maturation. Furthermore, it was assumed that the metabolic activity of the studied strains could influence the textural properties, oxidative stability, and qualitative characteristics of fermented beef sausages.

## 2. Materials and Methods

### 2.1. Materials

#### 2.1.1. LAB Strains

Three strains of LAB were used for the research treatment: *Lactiplantibacillus plantarum* S2A (GenBank accession OP784365), *Lactiplantibacillus pentosus* S4B (GenBank accession OP793706), and *Lactiplantibacillus plantarum* OP4 (GenBank accession OP793897). Strains S2A and S4B were isolated from organic beef hams, while strain OP4 was isolated from the production table of a meat processing plant. In a previous study, we showed that the given strains have antioxidant potential that can be used in the production of meat products. Of the twenty-one LAB strains, we selected three to test their activity in a meat product [18].

Bacterial isolates were stored in MRS (de Man, Rogosa, Sharpe) broth (Biomaxima, Lublin, Poland) with 20% glycerol addition (*v*/*v*; Merck, Darmstadt, Germany). To prepare bacterial cultures for sausage production, frozen isolates were revived by adding 0.1 mL of bacteria to 9.9 mL of fresh MRS broth. Next, 18 h bacterial cultures were prepared for the inoculation of the meat batter. MRS broth was centrifuged in an MPW-56 centrifuge (MPW Med. Instruments, Warsaw, Poland) at 6000 min^−1^ (3341× *g*) for 10 min. The remaining biomass was then washed three times with phosphate buffered saline (PBS, pH 7.2–7.6; Merck, Darmstadt, Germany), centrifuging the supernatant each time. Finally, the bacterial biomass was resuspended in 0.1 L of saline solution (0.9%; Merck, Darmstadt, Germany). The initial count of LAB in the biomass for strain S2A was 7.31 ± 0.15 log CFU/mL, for strain S4B was 7.02 ± 0.22 log CFU/mL, and for strain OP4 was 7.68 ± 0.08 log CFU/mL. The count of LAB was determined in accordance with Section 2.2.2.

#### 2.1.2. Fermented Beef Sausages

Four fermented beef sausage treatments were prepared (Table 1). The control sample (C) was produced without the addition of LAB cultures. The test samples, S2A, S4B, and OP4, were produced with the addition of LAB, *L. plantarum* S2A, *L. pentosus* S4B, or *L. plantarum* OP4 cultures, respectively. The estimated initial number of LAB in raw meat was 5 log CFU/g. The addition of starter cultures was used to standardize the fermentation process and reduce batch-to-batch variability.

The raw meat for sausage production was pre-cut, manually mixed with salt (NaCl 99.9%; 0.20 kg; Chempur, Piekary Śląskie, Poland) and stored at 2–4 °C for 48 h. The lean trimmings obtained from beef meat (M. *semimembranosus;* 10 kg) were ground in a meat grinder using 10 mm mesh. Glucose (highly purified D-glucose, 99.7%; Chempur) and the saline solution or bacterial biomass suspended in the saline solution were added to the ground meat, depending on the treatment. The meat and ingredients were mixed for 5 min in a mixer (Mainca LM 40, Barcelona, Spain). The sausage filling was stuffed into natural casings (pork intestines, diameter 26–28 mm) and hung on smoking trolleys. The sausages were formed into bars weighing approx. 75 g. The sausages were left in the production hall to dry their casings at 10–12 °C for 4 h. Subsequently, they were transferred to a fermentation and ripening chamber. The sausages were subjected to a two-stage process consisting of fermentation followed by ripening. During the first stage, the products were kept at 15–17 °C and 70–85% relative humidity for 14 days. On day 3 of this stage, the sausages were smoked (20–25 °C for 20 min) in a smoking chamber (KWP2/G, Rex-Pol, Chorzów, Poland). Smoking was carried out using cold smoke to impart sensory characteristics and limit the development of surface microbiota. After 14 days, the temperature was reduced to 10–12 °C, and the ripening process was continued until day 35. After completion of the process, the final products were cooled to 4–6 °C and vacuum-packed in multilayer polyamide and polyethylene film.

The produced beef sausages had an average water content of 39.6%, protein 39.6%, fat 12.2%, and carbohydrates 0.5%. The salt content was on average 5.5%.

### 2.2. Methods

#### 2.2.1. Sampling Procedures

Three independent production batches of beef sausages were conducted. The products were manufactured under industrial conditions at a meat processing plant operating under an organic production system (PL-EKO-01.616-0009988.2025.001). The plant does not use starter cultures in the production of meat products.

Analyses were conducted immediately after the packaged sausages arrived at the laboratory. The cold chain was maintained throughout transportation at 2 °C.

#### 2.2.2. Microbiological Analysis

All microbiological media were purchased from Biomaxima (Lublin, Poland). Total aerobic mesophilic count was determined according to ISO 4833-1:2013 [19] using PCA (Plate Count Agar) LAB-AGAR™. *Enterobacteriaceae* count was determined according to ISO 21528-2:2017 [20] using MacConkey LAB-AGAR™. Beta-glucuronidase-positive *Escherichia coli* count was determined according to ISO 16649-1:2018 [21] using TBX (Tryptone Bile X-glucuronide) LAB-AGAR™. The enumeration of mesophilic LAB was determined according to ISO 15214:1998 [22] using MRS (de Man, Rogosa, Sharpe) LAB-AGAR™. The enumeration of coagulase-positive staphylococci (*Staphylococcus aureus* and others) was determined according to ISO 6888-1:2021 [23] using Baird Parker LAB-AGAR™. The enumeration of yeasts and molds was determined according to ISO 21527-2:2008 [24] using Sabouraud Dextrose with Chloramphenicol LAB-AGAR™. The number of microorganisms is expressed as log CFU/g. Three different sausages were sampled for testing, and the tests were performed in six repetitions.

The presence of *Campylobacter* spp. was checked according to ISO 10272-1:2017 [25] using Bolton pre-management broth and Chromogenic *Campylobacter* LAB-AGAR™. The presence of *Salmonella* spp. was checked according to ISO 6579-1:2017 [26] using BPW (Buffered Peptone Water) and XLD (Xylose Lysine Deoxycholate) LAB-AGAR™. The presence of *Listeria* spp. was checked according to ISO 11290-1:2017 [27] using half-Fraser/Fraser broth for pre-enrichment and Chromogenic *Listeria* LAB-AGAR™. The result is expressed as the presence (pr) of or not detected (nd) bacteria in 25 g of product. Three different sausages were sampled for testing, and the tests were performed in three repetitions.

#### 2.2.3. Water Activity Measurements

Water activity was measured according to ISO 18787:2017 [28] using an Aqualab Pawkit DE201 (Aqualab, Warsaw, Poland). Calibration was performed prior to measurements using reference solutions provided by the manufacturer. Measurements were performed at 20 ± 1 °C. Three different sausages were sampled for testing, and the tests were performed in six repetitions.

#### 2.2.4. pH Measurements

pH was determined according to ISO 2917:1999 [29] using a SevenCompactTM S220 pH meter with a pH Sensor InLab^®^ Routine combination electrode (Mettler-Toledo GmbH, Greifensee, Switzerland). Calibration was performed prior to measurements using reference solutions provided by the manufacturer. Measurements were performed at 20 ± 1 °C. Three different sausages were sampled for testing, and the tests were performed in six repetitions.

#### 2.2.5. Oxidation–Reduction Potential Measurements

Oxidation–reduction potential was measured based on Nam & Ahn [30] using a SevenCompactTM S220 pH meter with an InLab Redox ORP electrode (Mettler-Toledo). Calibration was performed prior to measurements using reference solutions provided by the manufacturer. Measurements were performed at 20 ± 1 °C. The result is presented as mV. Three different sausages were sampled for testing, and the tests were performed in six repetitions.

#### 2.2.6. Analysis of Lipid Oxidation

Peroxide value was measured iodometrically according to ISO 3960:2017 [31]. The result is presented as meq O_2_ kg^−1^ fat. The tests were performed in six repetitions.

The thiobarbituric acid reactive substances index was measured based on the methodology of Pikul et al. [32]. Briefly, the content of substances reactive with thiobarbituric acid, primarily malondialdehyde (MDA), was measured. The intensity of the color produced by the reaction was measured using a U-2900 spectrophotometer (Hitachi, Tokyo, Japan) at a wavelength of 532 nm. The result is expressed as mg MDA kg^−1^ of product. Three different sausages were sampled for testing, and the tests were performed in six repetitions.

#### 2.2.7. Color Measurements

Instrumental color measurement was performed using the CIE Lab* system using a CR-300 Chroma Meter (Konica Minolta, Tokyo, Japan). Samples were prepared as fresh, 8 mm thick sausage slices left at room temperature for 20 min before measurement. The instrument was calibrated using a white standard (L* 99.18, a* −0.07, b* −0.05) provided by the manufacturer. An 8 mm diameter aperture and a standard 2° observer were used. The light source was a D65 illuminant and a pulsed xenon lamp. The measurements were performed at a temperature of 20 ± 1 °C. Three different sausages (six sausage cross-sections) were sampled for testing, and the tests were performed in eighteen repetitions.

#### 2.2.8. Fatty Acid Composition

The fatty acid profile was determined by gas chromatography with flame ionization detection (GC-FID) using an HP/Agilent 6890 II chromatograph (Hewlett-Packard Co., Palo Alto, Santa Clara, CA, USA), in accordance with ISO 12966-1:2014 [33] and Okoń et al. [34]. Results are presented as percentage/100 g of fat and a ratio. Three different sausages were sampled for testing, and the tests were performed in six repetitions.

#### 2.2.9. Texture Analysis

The textural properties of fermented beef sausages were analyzed with a texture analyzer (TA-XT. plus, Stable Micro Systems, Godalming Surrey, UK). All of the samples analyzed were cylindrical in shape, with a diameter of 20 mm and a height of 25 mm. The samples were compressed to 50% of their original height using a P/36R probe (Stable Micro Systems, UK) at a speed of 2 mm·s^−1^, with a load cell of 30 kg. Three different sausages were sampled for testing. Texture parameters were measured in six replicates for each sample, and the results were recorded.

#### 2.2.10. Free Amino Acid Content

Free amino acid content was analyzed according to the method developed by Świder et al. [35] with minor modifications. A detailed description of the modified version of the method in terms of sample preparation and ultra-high performance liquid chromatography–high-resolution mass spectrometry (UPLC–HRMS, Q Exactive Orbitrap Focus MS, Thermo Fisher Scientific, Waltham, MA, USA) parameters is described in the article by Chmiel et al. [36]. Three different sausages were sampled for testing, and the tests were performed in twelve repetitions.

#### 2.2.11. Statistical Analysis

The obtained results are presented as mean and standard deviation. Data were statistically analyzed using one-way analysis of variance (ANOVA) to assess the significance of differences between the study groups. If a significant effect of a factor was observed, a Tukey post hoc test was used, allowing for comparison of means between all pairs of groups. Differences were considered statistically significant at a significance level of *p* < 0.05. All calculations were performed using Statistica 13.1 (TIBCO Software Inc., Palo Alto, USA).

## 3. Results and Discussion

### 3.1. Microbiological Analysis

Table 2 presents the results of the microbiological analysis. The total aerobic mesophilic count was found to be high, ranging from 7.48 to 8.16 log CFU/g, with no differences between treatments (*p* > 0.05). Treatments C and S4B had low *Enterobacteriaceae* counts (2.03–2.46 log CFU/g), while treatments S2A and OP4 had counts below the detection limit (<1.00 log CFU/g). *E. coli* was not detected in any of the samples. All tested treatments were characterized by high mesophilic LAB counts (7.68–8.20), with no differences between treatments (*p* > 0.05). Coagulase-positive staphylococci were observed in all sausages (3.35–4.26 log CFU/g), with no *S. aureus*. Significantly, the lowest number of staphylococci was found in treatments S4B and OP4 (3.35–3.53 log CFU/g; *p* < 0.05). A significantly higher number of yeasts and molds was found in sausages with the addition of LAB (3.08–3.14 log CFU/g; *p* < 0.05). The presence of *Campylobacter* spp., *Salmonella* spp., and *Listeria* spp. was not observed in any of the tested treatments.

Maturing sausages, despite the use of fermentation and drying processes, may provide an environment conducive to the survival or growth of some spoilage and pathogenic bacteria [37,38,39]. The presence of microorganisms such as *Enterobacteriaceae*, *E. coli*, and *Pseudomonas* is responsible for meat spoilage and impacts product safety [39]. According to the Scientific Opinion of the EFSA (European Food Safety Authority) Panel on Biological Hazards [40], the microbiological hazards found in maturing meats are Shiga-toxin-producing *E. coli* (especially beef), *Salmonella* spp., *S. aureus*, *L. monocytogenes*, enterotoxigenic *Yersinia* spp., *Campylobacter* spp., *Clostridium* spp., and the molds *Aspergillus* spp. and *Penicillium* spp. High total aerobic mesophilic counts in maturing sausages are typical and usually result from the presence of desirable fermentative microbiota, long maturation, and the nature of the raw material [41]. Furthermore, sausages are a product tested together with their casing, where the presence of yeasts and molds increases the total number of microorganisms. An interesting phenomenon observed is the LAB count and the count of yeasts and molds. A significantly lower LAB count and a significantly higher yeast and mold count were observed in the treatments with starter cultures (*p* < 0.05). This could be due to several factors: the starters added to the batter did not adapt adequately to the environment and the indigenous microbiota dominated the environment, or increased competition among the microbiota allowed yeast and mold growth. Compounds formed as part of metabolic changes in the meat were more easily absorbed by yeasts and molds [42]. The presence of yeasts and molds in maturing sausages is not always undesirable, as selected surface microorganisms can participate in shaping the aroma, taste, and oxidative stability of the product [41,42]. Additionally, the reduction in water activity during maturation could have limited the survival of some starter strains. A study by Barcenilla et al. [37] demonstrated that LAB predominate in the final products, while they are present in low numbers on surfaces and in raw materials.

Although *Staphylococcus aureus* was not specifically detected in the products, the authors estimated the number of coagulase-positive staphylococci to be too high to perform a sensory analysis of the products. It is uncertain whether this number, along with the relatively high pH of the products, is sufficient to produce enterotoxins. In this case, without testing for the presence of enterotoxins in the product, the health safety of the products cannot be ensured, although, taking into account Commission Regulation (EC) No 1441/2007 [43], sensorial testing is not required. In meat processing plants, the occurrence of coagulase-positive *Staphylococcus* spp. is common [44]. High numbers of coagulase-positive staphylococci in raw-cured beef sausages may be due to several factors related to both the quality of the raw material and the production and curing conditions. The main source of these microorganisms is usually meat and production personnel, as staphylococci, especially *Staphylococcus* spp., constitute the natural microflora of the skin and mucous membranes of humans and animals [45]. Raw-cured sausages are characterized by a lack of high-temperature heat treatment, so microorganisms present in the raw material are not eliminated during the technological process. Furthermore, the initial stages of curing, involving relatively high water activity, nutrient availability, and moderate fermentation temperature, may favor the proliferation of staphylococci [45].

The obtained results indicate that the use of autochthonous starter cultures influenced the dynamics of the microbiota of maturing beef sausages, but this effect depended on the adaptability of individual strains to the product environment.

### 3.2. Water Activity, pH, Oxidation–Reduction Potential Measurements, Analysis of Lipid Oxidation

Our investigation determined that there are no significant differences in the basic composition and water activity of the sausages examined (*p* > 0.05) (Table 3). The pH value ranged from 5.42 to 5.82, and the sausages differed significantly from each other (*p* < 0.05). Interestingly, the sausages with the addition of bacteria (S2A, S4B, and OP4) were characterized by a higher pH value (5.69, 5.77, and 5.82, respectively) than the control (5.42). During the fermentation of sausages with the addition of LAB, the environment could be rapidly acidified (due to the production of organic acids, mainly lactic acid), and then the pH could increase during ripening. Another reason for the high pH could be the low activity or poor adaptation of the starter cultures to the process conditions, as well as the activity of microbiota competitive with LAB. In the treatments inoculated with LAB, higher yeast and mold counts were observed, which may have affected the acidification process [46].

The oxidation–reduction potential of the control group (C) showed a significantly higher level (between 15 and 31 mV) compared to the other treatments, as measured directly after the ripening process (*p* < 0.05). A decrease in redox potential promotes electron donation and the elimination of free radicals, which suggests that the potential may indicate the presence of antioxidant compounds. The results suggest the potential formation of low-molecular-weight protein compounds, designated as S2A, S4B, and OP4, during the various treatments, which influenced the redox potential. This change is also confirmed by the observed higher pH values. Some LAB strains have been reported to exhibit antioxidant activity associated with the production of enzymes such as manganese-containing pseudocatalase and glutathione peroxidase [47,48]. The production of these antioxidant enzymes by specific strains of LAB may have contributed to the decrease in the redox potential observed in the sausages studied. Although treatments inoculated with LAB showed lower oxidation–reduction potential values than the control, this was not fully reflected in the peroxide value and TBARS results. Oxidation–reduction potential describes the general redox environment of the product, whereas peroxide value and TBARS are direct indicators of lipid oxidation. Therefore, these parameters may not always show a direct correlation. The higher peroxide and TBARS values observed in treatment S2A suggest that lipid oxidation progressed despite the lower oxidation–reduction potential. This may be related to differences in microbial metabolism and the complex biochemical changes occurring during sausage ripening.

The peroxide value serves as an indicator of lipid oxidation in meat products, reflecting the rate at which primary lipid oxidation products are generated. In the initial stages of oxidation, hydroperoxide levels rise as their formation outpaces decomposition. However, due to the instability of these compounds, later stages reveal greater decomposition than formation, resulting in a decrease in hydroperoxide content, as reflected by a lower peroxide value [49]. Table 3 demonstrates that sausages containing the S4B strain exhibited significantly lower peroxide value compared to other samples. This is likely attributed to the high levels of protein breakdown products, which can scavenge lipid radicals and thereby reduce lipid oxidation. Furthermore, these S4B sausages also showed a low redox potential and lower TBARS values.

To understand how well different food preservation methods work and how they affect food quality and safety, it is helpful to look at the secondary compounds created during these processes [46]. One important measurement in this analysis is the TBARS value, which indicates how much lipids have oxidized in food products. Higher TBARS values often mean stronger off-flavors, suggesting that as lipids oxidize, foods can develop unpleasant tastes that may reduce consumer acceptance. The sensory panel’s threshold for detecting rancid taste and odor is 2.00–2.50 mg MDA kg^−1^ of product [49]. None of our treatments exceeded this threshold. The statistically significant lowest TBARS value was recorded in sample OP4 (*p* < 0.05). This treatment also had the lowest redox potential, indicating good quality and high oxidative stability.

### 3.3. Color Measurements

No statistically significant differences were found between the treatments in the L* color brightness parameter (*p* > 0.05) (Table 4). The L* value ranged from 37.41 in treatment OP4 to 41.29 in treatment S4B, meaning that S4B was slightly brighter than the others. Treatment S4B also had a significantly higher red color (a*) compared to the other treatments with LAB addition but did not differ significantly from the control treatment. The b* parameter was similar, being significantly higher in treatments C and S4B and significantly lower in S2A and OP4. The color angle (h°) and color saturation (C*) in the tested treatments were consistent with the obtained results. Favorable h° and C* parameters, similar to those of the control sample, may indicate the protective effect of the S4B strain on the stability of dyes in the product. Analysis of the total color difference (ΔE*) showed that inoculating LAB into the meat matrix significantly modified the color profile in all experimental groups compared to the control treatment. The recorded ΔE* values ranged from 6.05 to 7.30. According to the literature [50,51], this represents a difference that is clearly noticeable to the average observer (ΔE* threshold >5.0). The highest ΔE* value (7.30) was recorded in treatment S4B. While this parameter mathematically indicates the greatest deviation from the standard, its correlation with high red color (a*) and saturation (C*) suggests positive color intensification rather than degradation. This phenomenon can be explained by the ability of the selected LAB strains to reduce metmyoglobin or stabilize oxymyoglobin [52]. At low water activity, this leads to the desired deep red color. On the other hand, the lower ΔE* values in treatments S2A (6.72) and OP4 (6.05) do not suggest a higher level of similarity to the standard in terms of quality. This is probably due to a simultaneous decrease in the a* parameter and L* brightness, which brought these samples closer to the control in terms of the total color vector but indicated the progressive oxidation of heme pigments. This phenomenon is often described as color fading, which is typical of meat with reduced oxidative stability [53].

### 3.4. Fatty Acid Composition

Saturated fatty acids (SFAs) were the most dominant in beef sausages, where the highest content was found in products S2A and S4B (52.25 and 52.90 g/100 g of fat) and the lowest in the control (46.45 g/100 g of fat) (Table 5). Palmitic acid (C16:0) and stearic acid (C18:0) constituted the majority of SFAs. SFAs are known to have a negative impact on cardiovascular disease [54]. Studies show they may also be a significant risk factor for type 2 diabetes and insulin resistance [55]. According to nutritional recommendations, the consumption of SFAs should be limited to avoid exceeding 10% of total energy intake, as recommended by the World Health Organization (WHO) [56]. However, the highest content of polyunsaturated fatty acids (PUFAs) was present in the S2A and OP4 sausages (7.40 and 7.25 g/100 g of fat). PUFAs are desirable for health, including linoleic acid (C18:2) and alpha-linoleic acid (C18:3) [57]. Samples containing S2A and OP4 strains exhibited the highest PUFA content in the fatty acid pool. This phenomenon may be due to two reasons. LAB and their metabolites may possess antioxidant properties that contribute to the oxidation inhibition of unsaturated fatty acids. Furthermore, some LAB possess the desaturase enzyme and can convert free saturated fatty acids into unsaturated ones [58]. According to nutritional recommendations, the ratio of n-6 to n-3 fatty acids should be 1:1 to 5:1 [59]. In all of the analyzed products, the ratios were within limits. Monounsaturated fatty acids (MUFAs), especially oleic acid (C18:1), were the most abundant among the fatty acids in the analyzed samples (the share was 28.05 for S2A and 35.55 g/100 g of fat for C). The average content of MUFAs was significantly lower in sausages with LAB (*p* < 0.05). The content of SFAs, MUFAs, and PUFAs was statistically different among samples.

LAB’s lipid metabolism has a significant impact on the final product quality. Bacterial lipase leads to hydrolysis and results in free fatty acid and glyceride formation and further oxidation. Those compounds provide a carbon source for LAB [60]. In the previous study [61] it was found that pork sausages with the addition of nitrates had a higher share of PUFAs; however, it was also found that in comparison to control samples without the addition of bacteria but only treated with salt, there was a lower content of these nutritionally valuable fatty acids in comparison to sausages with the *P. pentosaceus* KL14 strain. Moreover, it was also found that sausages with LAB had higher share of MUFAs compared to the control sample.

In the research of Wójciak et al. [62], no significant differences were found between the control sample and sausages fermented by probiotic LAB strains—*L. casei* LOCK 0900, *L. casei* LOCK 0908, and *L. paracasei* LOCK 0919.

Rumen bacteria and some LAB are known for the transformation of linoleic acid to steric acid, where CLA (conjugated linoleic acid; C18:2 c9t11) and vaccenic acid (C18:1trans) are formed as intermediate products [63]. CLA is known for its potential pro-health properties [64]. However, C18:1trans should be avoided due to its negative impact on health, especially the cardiovascular system [65]. Although the CLA content did not increase significantly in the analyzed fermented sausages, C18:1 trans increased. In the control sample, the content of this acid was 1.5%, while in the fermented sausages it ranged from 2.1% for the sample with OP4 to 2.4% for the sample with the S4B strain (Table 5). In the study of Özer & Kılıç [64], during optimal fermentation of ground beef by *L. plantarum* DSM 2601 and *L. plantarum* AB20–961 strains, the CLA increased from 3.73 before fermentation to 42.32 and 7.65 mg/g fat, respectively. In the case of C18:1 trans, the increase was from 9.23 to 18.29 and 14.87 mg/g fat, respectively.

### 3.5. Texture Analysis

Texture is one of the most important characteristics determining the quality and consumer acceptance of food products, including processed meats. It is a complex characteristic, dependent on the product’s structure, chemical composition, and physical properties. Texture encompasses parameters such as hardness, adhesiveness, cohesiveness, gumminess, chewiness, and elasticity, the values of which may vary depending on the product type [66]. In fermented sausages, textural properties can change due to lower pH, denaturation and aggregation of myofibrillar proteins, and changes occurring during maturation and drying. Myofibrillar proteins form a gel structure, which can increase the sausage’s hardness, gumminess, or chewiness. Textural properties also depend largely on moisture content and loss [67].

The hardness of the tested samples ranged from 288.01 to 338.02 N (Table 6). A significantly lower value of this parameter, compared to the other samples, was found in the treatment containing *L. plantarum* OP4. The observed changes in textural parameters could be due to changes in moisture content and the water-binding capacity of muscle proteins. A lower water content typically leads to increased hardness and chewiness of meat products, as water acts as a plasticizer in the structure of muscle proteins. Furthermore, a decrease in pH can cause the denaturation of muscle proteins and changes in the organization of the protein gel structure, thus affecting the cohesiveness of the product [68].

Cohesiveness did not change significantly. Similar relationships were observed by Cavalheiro et al. [69] in studies of fermented dry sausages inoculated with *Enterococcus faecium* CECT 410 and by Ye et al. [66] in studies using mixed starter cultures.

### 3.6. Free Amino Acid Content

The effect of applied starter cultures on the analyzed free amino acid (FAA) concentrations in fermented beef sausages is summarized in Table 7. Total FAA content in the final product was significantly lower in the samples inoculated with the selected starter cultures in comparison to the control, whereby this effect was stronger for S4B and OP4 strains (total FAA content lower by 29.7% and 33.5%, respectively, compared to the control) than for S2A (10.1% lower total FAA). Concentrations of most analyzed FAAs were significantly lower in the S4B and OP4 treatments in comparison to the S2A or control treatment. Ornithine and glutamine were an exception; their concentrations were the lowest in control samples. Since both these FAAs represent precursors (direct and indirect, respectively) for putrescine formation, they could be utilized by autochthonous microorganisms present in control samples in the formation of this biogenic amine (BA). Also, the concentration of arginine, another indirect precursor of putrescine, was significantly lower in the control and OP4 treatments, while the concentration of tryptophan, a precursor of tryptamine, was the lowest in the S2A and OP4 treatments. The observed differences can suggest that the mentioned BAs were produced; however, additional analysis of BA content, as well as thorough characterization of the microorganisms present in the samples, could facilitate a precise explanation of the obtained results. Glutamic acid, lysine, and alanine were the most abundant FAAs in all tested samples. Concentrations of these FAAs were in the ranges 1.9–2.6 g/kg, 1.4–1.9 g/kg, and 1.1–1.5 g/kg, respectively. Similarly, these three amino acids prevailed in fermented pork sausages, both control and inoculated with single or mixed starter cultures, as reported by Aro et al. [70]. In the study conducted by this research group, application of *Staphylococcus xylosus* alone resulted in the highest total FAA content, while samples inoculated with a mix of *P. pentosaceus* and *S. xylosus* contained the lowest total FAA concentration among the six tested treatments (without starter cultures, *L. sakei*, *S. carnosus*, *S. xylosus*, *L. sakei* + *S. carnosus*, or *P. pentosaceus* + *S. xylosus*) [70]. Candogan et al. [71] examined four individual strains belonging to *P. acidilactici*, *L. curvatus*, *L. sake*, or *Str. griseus* species regarding changes in FAA concentration in fermented beef sausages. Total FAA content did not differ significantly between the tested treatments; however, some differences were observed between respective compounds. Alanine, leucine, glutamic acid, glutamine, lysine, and ornithine predominated among all tested FAAs [70]. In the research conducted by Domínguez et al. [72], control samples of dry-cured foal sausages contained a significantly lower total FAA content compared to samples inoculated with the following starter culture mixes: *S. carnosus* + *S. xylosus* + *P. pentosaceus*, *D. hansenii* + *S. xylosus*, or *P. pentosaceus* + *S. xylosus*. Leucine, phenylalanine, and cysteine were the most abundant among the analyzed FAAs [72]. Both the endogenous enzymes of meat and those produced by applied microorganisms are involved in the proteolytic reactions that occur during the production of fermented sausages and ultimately lead to the release of amino acids [71]. Owing to the different proteolytic activity exhibited by individual microorganisms, the application of various microbial strains enables obtaining varied products in terms of amino acid content. Since amino acids represent precursors for microbial and chemical reactions leading to the formation of compounds such as, BAs or volatile aroma compounds, the selection of starter cultures is crucial considering the safety and sensory properties of the final product [73,74].

Fermentation is a complex process affected by numerous factors, such as autochthonous and added microorganism characteristics, raw material quality, type of food additives used, and manufacturing conditions. Thus, each potential starter culture intended to be applied should first be examined in the target matrix to assess its influence on the quality of the final product.

## 4. Conclusions

The study results indicate that indigenous strains of LAB isolated from meat demonstrate significant potential as starter cultures in the production of fermented beef sausages. Their use significantly improved product quality, promoting the maintenance of high numbers of desirable fermenting bacteria while maintaining an appropriate amount of yeasts and molds, as well as the overall microbiological safety of the product. The addition of bacteria also determined the physicochemical and technological properties of the sausages, including color, texture, and lipid and amino acid profile. The observed changes in fatty acid composition, particularly the increased content of PUFAs, may indicate a potential improvement in the product’s nutritional value. Furthermore, differences in the level of lipid oxidation and the intensity of proteolytic transformations indicate the varying impact of individual strains on oxidative stability. The use of LAB strains allows for the reduction in or complete elimination of nitrate addition in the production of meat products, as it enables color development. The *Lactiplantibacillus plantarum* OP4 strain demonstrated particularly favorable properties, characterized by the lowest level of lipid oxidation while maintaining desirable quality parameters. In summary, the use of selected, native LAB strains is a promising strategy in the production of fermented beef sausages, enabling effective control of the fermentation process, shaping the product’s quality characteristics, and effectively overcoming the limitations associated with beef processing. At the same time, the relatively high pH values and the number of coagulase-positive staphylococci indicate the need for further optimization of fermentation conditions and the use of starter cultures to improve the microbiological safety of fermented beef sausages. However, the obtained results confirm the potential of the tested native LAB strains for use as starter cultures in the production of fermented meat products.

## Figures and Tables

**Table 1:** Raw material composition of the produced fermented beef sausages.

Component	Treatment			
	C	S2A	S4B	OP4
Beef meat *M. semimembranosus* (kg)	10.00	10.00	10.00	10.00
Glucose (kg)	0.05	0.05	0.05	0.05
NaCl (kg)	0.20	0.20	0.20	0.20
Saline solution (L)	0.10	-	-	-
Saline solution + S2A (L)	-	0.10	-	-
Saline solution + S4B (L)	-	-	0.10	-
Saline solution + OP4 (L)	-	-	-	0.10

C, control treatment, without starter cultures; S2A, treatment with *L. plantarum* S2A; S4B, treatment with *L. pentosus* S4B; OP4, treatment with *L. plantarum* OP4.

**Table 2:** Microbiological evaluation of the tested fermented beef sausages.

Parameter	Treatment			
	C	S2A	S4B	OP4
Total aerobic mesophilic count (log CFU/g)	8.16 ± 0.28 ^a^	7.94 ± 0.14 ^a^	7.48 ± 0.55 ^a^	7.78 ± 0.26 ^a^
*Enterobacteriaceae* (log CFU/g)	2.46 ± 0.04 ^b^	<1.00 ^a^	2.03 ± 0.00 ^b^	<1.00 ^a^
Beta-glucuronidase-positive *Escherichia coli* (log CFU/g)	<1.00 ^a^	<1.00 ^a^	<1.00 ^a^	<1.00 ^a^
Mesophilic lactic acid bacteria (log CFU/g)	8.20 ± 0.17 ^a^	7.68 ± 0.59 ^a^	8.04 ± 0.44 ^a^	8.01 ± 0.50 ^a^
Coagulase-positive staphylococci (*S. aureus* and other species) (log CFU/g)	4.14 ± 0.13 ^b,^*	4.26 ± 0.08 ^b,^*	3.35 ± 0.27 ^a,^*	3.53 ± 0.09 ^a,^*
Yeasts and molds (log CFU/g)	2.11 ± 0.22 ^a^	3.08 ± 0.08 ^b^	3.10 ± 0.01 ^b^	3.14 ± 0.08 ^b^
*Campylobacter* spp.	nd	nd	nd	nd
*Salmonella* spp.	nd	nd	nd	nd
*Listeria* spp., including *L. monocytogenes*	nd	nd	nd	nd

C, control treatment, without starter cultures; S2A, treatment with *L. plantarum* S2A; S4B, treatment with *L. pentosus* S4B; OP4, treatment with *L. plantarum* OP4; <1.00, below the detection limit; * no *Staphylococcus aureus* was recorded; nd—not detected. Different lowercase letters in the same row indicate statistically significant differences between groups according to Tukey’s test (*p* < 0.05).

**Table 3:** Physicochemical parameters and evaluation of lipid oxidation of the tested fermented beef sausages.

Parameter	Treatment			
	C	S2A	S4B	OP4
Water activity	0.78 ± 0.01 ^a^	0.79 ± 0.01 ^a^	0.79 ± 0.01 ^a^	0.76 ± 0.01 ^a^
pH	5.42 ± 0.01 ^a^	5.69 ± 0.01 ^b^	5.77 ± 0.01 ^b^	5.82 ± 0.01 ^b^
Oxidation–reduction potential (mV)	469.87 ± 7.88 ^b^	454.40 ± 4.15 ^a^	441.03 ± 8.67 ^a^	438.07 ± 1.80 ^a^
Peroxide value (meq O_2_ kg^−1^ of fat)	1.76 ± 0.02 ^b^	1.98 ± 0.01 ^c^	1.65 ± 0.03 ^a^	1.86 ± 0.02 ^b^
Thiobarbituric acid reactive substances (mg MDA kg^−1^ of product)	1.536 ± 0.018 ^b^	1.784 ± 0.011 ^c^	1.419 ± 0.009 ^ab^	1.340 ± 0.063 ^a^

C, control treatment, without starter cultures; S2A, treatment with *L. plantarum* S2A; S4B, treatment with *L. pentosus* S4B; OP4, treatment with *L. plantarum* OP4. Different lowercase letters in the same row indicate statistically significant differences between groups according to Tukey’s test (*p* < 0.05).

**Table 4:** Color parameters of the tested fermented beef sausages.

Parameter	Treatment			
	C	S2A	S4B	OP4
L*	37.45 ± 4.71 ^a^	39.21 ± 3.65 ^a^	41.29 ± 4.53 ^a^	37.41 ± 1.92 ^a^
a*	6.54 ± 1.81 ^b^	4.03 ± 1.63 ^a^	6.15 ± 2.30 ^b^	3.99 ± 1.92 ^a^
b*	6.61 ± 1.17 ^b^	4.75 ± 1.51 ^a^	5.78 ± 1.81 ^b^	4.43 ± 0.87 ^a^
hº	46.04 ± 7.19 ^a^	50.6 ± 9.80 ^b^	44.3 ± 8.90 ^a^	50.0 ± 9.80 ^b^
C*	9.36 ± 1.86 ^b^	6.43 ± 1.97 ^a^	8.42 ± 2.48 ^b^	6.06 ± 1.93 ^a^
ΔE*	-	6.72 ± 2.90 ^b^	7.30 ± 4.43 ^b^	6.05 ± 2.48 ^a^

C, control treatment, without starter cultures; S2A, treatment with *L. plantarum* S2A; S4B, treatment with *L. pentosus* S4B; OP4, treatment with *L. plantarum* OP4. Different lowercase letters indicate statistically significant differences between groups according to Tukey’s test (*p* < 0.05).

**Table 5:** Fatty acid profile of the tested fermented beef sausages.

Parameter (g/100 g of Fat)	Treatment			
	C	S2A	S4B	OP4
C12:0	0.10 ± 0.00 ^a^	0.15 ± 0.07 ^a^	0.10 ± 0.00 ^b^	0.10 ± 0.00 ^a^
C14:0	2.55 ± 0.07 ^a^	4.55 ± 0.49 ^b^	3.45 ± 0.21 ^b^	2.90 ± 0.28 ^a^
C14:1	0.80 ± 0.00 ^a^	1.15 ± 0.07 ^a^	0.95 ± 0.07 ^a^	0.80 ± 0.00 ^a^
C15:0 br	0.30 ± 0.00 ^a^	0.35 ± 0.07 ^a^	0.40 ± 0.00 ^a^	0.30 ± 0.00 ^a^
C15:0	0.55 ± 0.07 ^a^	0.70 ± 0.00 ^a^	0.80 ± 0.00 ^a^	0.65 ± 0.07 ^a^
C15:1	0.25 ± 0.07 ^a^	0.30 ± 0.00 ^a^	0.30 ± 0.00 ^a^	0.30 ± 0.00 ^a^
C16:0	24.25 ± 0.07 ^a^	25.65 ± 0.49 ^a^	25.40 ± 0.57 ^a^	23.85 ± 0.07 ^a^
C16:1	3.65 ± 0.07 ^a^	3.50 ± 0.00 ^a^	3.35 ± 0.21 ^a^	3.25 ± 0.07 ^a^
C17:0 br	1.15 ± 0.07 ^a^	1.20 ± 0.00 ^a^	1.30 ± 0.00 ^a^	1.30 ± 0.00 ^a^
C17:0	1.05 ± 0.07 ^a^	1.10 ± 0.00 ^a^	1.40 ± 0.00 ^a^	1.25 ± 0.07 ^a^
C17:1	0.60 ± 0.00 ^a^	0.50 ± 0.00 ^a^	0.50 ± 0.00 ^a^	0.55 ± 0.07 ^a^
C18:0	17.45 ± 0.21 ^a^	19.55 ± 0.07 ^a^	21.05 ± 0.49 ^a^	19.75 ± 0.64 ^a^
C18:1trans	1.50 ± 0.14 ^a^	2.25 ± 0.07 ^b^	2.40 ± 0.00 ^b^	2.10 ± 0.14 ^b^
C18:1cis 9	35.55 ± 0.07 ^b^	28.05 ± 0.78 ^a^	29.05 ± 0.07 ^a^	32.20 ± 0.98 ^b^
C18:1cis 11	1.80 ± 0.14 ^a^	1.60 ± 0.00 ^a^	1.50 ± 0.00 ^a^	1.55 ± 0.07 ^a^
C18:1 others	1.20 ± 0.00 ^a^	1.30 ± 0.00 ^a^	1.25 ± 0.07 ^a^	1.25 ± 0.07 ^a^
C18:2 n6	3.55 ± 0.21 ^a^	3.80 ± 0.28 ^b^	3.40 ± 0.28 ^a^	3.95 ± 0.35 ^b^
C18:3 n3	0.70 ± 0.00 ^a^	0.85 ± 0.07 ^a^	0.80 ± 0.00 ^a^	0.75 ± 0.07 ^a^
C18:2c9t11 CLA	0.35 ± 0.07 ^a^	0.40 ± 0.00 ^a^	0.40 ± 0.00 ^a^	0.30 ± 0.00 ^a^
C20:0	0.20 ± 0.00 ^a^	0.20 ± 0.00 ^a^	0.30 ± 0.00 ^a^	0.20 ± 0.00 ^a^
C20:1	0.40 ± 0.00 ^a^	0.30 ± 0.00 ^a^	0.30 ± 0.00 ^a^	0.30 ± 0.00 ^a^
C20:3n3	0.20 ± 0.00 ^a^	0.20 ± 0.00 ^a^	0.15 ± 0.07 ^a^	0.25 ± 0.07 ^a^
C20:4n6	0.95 ± 0.07 ^a^	1.20 ± 0.14 ^a^	0.75 ± 0.07 ^a^	1.15 ± 0.21 ^a^
C20:5 EPA	0.20 ± 0.00 ^a^	0.25 ± 0.07 ^a^	0.15 ± 0.07 ^a^	0.20 ± 0.00 ^a^
C22:4n6	0.10 ± 0.00 ^a^	0.10 ± 0.00 ^a^	0.10 ± 0.00 ^a^	0.10 ± 0.00 ^a^
C22:5n3	0.40 ± 0.00 ^a^	0.50 ± 0.00 ^a^	0.35 ± 0.07 ^a^	0.45 ± 0.07 ^a^
C22:6 DHA	0.10 ± 0.00 ^a^	0.10 ± 0.00 ^a^	0.10 ± 0.00 ^a^	0.10 ± 0.00 ^a^
SFA	46.45 ± 0.49 ^a^	52.25 ± 1.20 ^c^	52.90 ± 0.28 ^c^	49.00 ± 1.13 ^b^
MUFA	45.35 ± 0.21 ^c^	38.65 ± 0.64 ^a^	39.30 ± 0.28 ^a^	42.00 ± 1.84 ^b^
PUFA	6.55 ± 0.21 ^a^	7.40 ± 0.57 ^b^	6.20 ± 0.57 ^a^	7.25 ± 0.78 ^b^
n-3	1.40 ± 0.00 ^a^	1.70 ± 0.14 ^b^	1.40 ± 0.14 ^a^	1.50 ± 0.14 ^a^
n-6	4.80 ± 0.28 ^a^	5.30 ± 0.42 ^b^	4.40 ± 0.42 ^a^	5.45 ± 0.64 ^b^
n-9	35.55 ± 0.07 ^c^	28.05 ± 0.78 ^a^	29.05 ± 0.07 ^a^	32.20 ± 1.98 ^b^
n-6/n-3 ratio (-)	3.43 ± 0.02 ^a^	3.12 ± 0.00 ^a^	3.14 ± 0.00 ^a^	3.63 ± 0.01 ^a^

C, control treatment, without starter cultures; S2A, treatment with *L. plantarum* S2A; S4B, treatment with *L. pentosus* S4B; OP4, treatment with *L. plantarum* OP4; CLA, conjugated linoleic acid; EPA, eicosapentaenoic acid; DHA, docosahexaenoic acid; SFA, saturated fatty acid; MUFA, monounsaturated fatty acid; PUFA, polyunsaturated fatty acid; Different lowercase letters indicate statistically significant differences between groups according to Tukey’s test (*p* < 0.05).

**Table 6:** Texture parameters of the tested fermented beef sausages.

Parameter	Treatment			
	C	S2A	S4B	OP4
Hardness (N) Force 1	337.16 ± 61.21 ^b^	338.02 ± 21.41 ^b^	320.94 ± 44.75 ^b^	288.01 ± 24.00 ^a^
Hardness (N) Force 2	204.63 ± 38.19 ^a^	324.95 ± 31.18 ^b^	257.85 ± 20.89 ^a^	317.36 ± 28.22 ^b^
Adhesiveness (N·s)	−30.51 ± 19.24 ^a^	−34.47 ± 19.10 ^a^	-24.88 ± 15.21 ^a^	−11.60 ± 5.63 ^a^
Springiness	0.71 ± 0.11 ^b^	0.62 ± 0.03 ^a^	0.60 ± 0.02 ^a^	0.63 ± 0.05 ^a^
Cohesiveness	0.59 ± 0.04 ^a^	0.57 ± 0.02 ^a^	0.57 ± 0.03 ^a^	0.54 ± 0.02 ^a^
Chewiness (N)	85.30 ± 20.06 ^a^	114.04 ± 9.87 ^b^	88.09 ± 9.13 ^a^	109.67 ± 21.52 ^b^
Resilience	0.19 ± 0.02 ^a^	0.19 ± 0.01 ^a^	0.19 ± 0.00 ^a^	0.19 ± 0.01 ^a^

C, control treatment, without starter cultures; S2A, treatment with *L. plantarum* S2A; S4B, treatment with *L. pentosus* S4B; OP4, treatment with *L. plantarum* OP4; Different lowercase letters indicate statistically significant differences between groups according to Tukey’s test (*p* < 0.05).

**Table 7:** Free amino acid content in the tested fermented beef sausages.

Parameter	Treatment			
	C	S2A	S4B	OP4
Asparagine (mg/kg)	143.97 ± 27.18 ^b^	223.23 ± 17.50 ^c^	134.42 ± 5.49 ^b^	68.03 ± 9.29 ^a^
Arginine (mg/kg)	135.60 ± 25.84 ^a^	349.53 ± 56.40 ^c^	220.90 ± 22.41 ^b^	156.61 ± 25.16 ^a^
Glutamine (mg/kg)	51.04 ± 9.25 ^a^	216.39 ± 27.64 ^c^	203.53 ± 9.96 ^c^	122.04 ± 20.66 ^b^
Serine (mg/kg)	803.26 ± 154.00 ^c^	650.17 ± 34.69 ^b^	547.51 ± 22.57 ^a^	542.85 ± 38.98 ^a^
Aspartic Acid (mg/kg)	659.57 ± 131.63 ^b^	223.08 ± 44.80 ^a^	211.84 ± 38.90 ^a^	206.66 ± 10.55 ^a^
Glutamic Acid (mg/kg)	2444.27 ± 498.33 ^b^	2614.97 ± 371.23 ^b^	1952.54 ± 102.06 ^a^	1869.90 ± 195.48 ^a^
Threonine (mg/kg)	732.63 ± 138.58 ^b^	604.36 ± 93.35 ^b^	444.66 ± 26.67 ^a^	451.05 ± 37.46 ^a^
Glycine (mg/kg)	591.52 ± 107.46 ^b^	467.86 ± 40.89 ^a^	400.66 ± 20.12 ^a^	393.25 ± 35.55 ^a^
Alanine (mg/kg)	1414.11 ± 272.85 ^b^	1455.95 ± 209.85 ^b^	1064.06 ± 42.19 ^a^	1055.45 ± 111.91 ^a^
Proline (mg/kg)	445.90 ± 88.56 ^b^	472.72 ± 55.25 ^b^	412.73 ± 17.49 ^a^	382.60 ± 32.97 ^a^
Methionine (mg/kg)	870.87 ± 169.36 ^b^	846.32 ± 167.31 ^b^	567.80 ± 29.06 ^a^	530.68 ± 46.64 ^a^
Valine (mg/kg)	1161.46 ± 258.03 ^b^	919.04 ± 103.45 ^b^	726.56 ± 34.27 ^a^	685.93 ± 63.33 ^a^
Tryptophan (mg/kg)	156.77 ± 38.05 ^b^	114.42 ± 14.49 ^a^	139.30 ± 10.79 ^b^	121.30 ± 14.90 ^a^
Phenylalanine (mg/kg)	1079.97 ± 221.11 ^c^	843.68 ± 65.41 ^b^	692.13 ± 34.84 ^a^	645.60 ± 55.12 ^a^
Isoleucine (mg/kg)	799.78 ± 173.80 ^c^	659.88 ± 64.06 ^b^	526.56 ± 25.46 ^a^	487.89 ± 42.96 ^a^
Leucine (mg/kg)	979.54 ± 188.53 ^c^	765.22 ± 35.44 ^b^	669.67 ± 25.85 ^a^	614.17 ± 53.69 ^a^
Ornithine (mg/kg)	176.95 ± 23.24 ^a^	304.98 ± 80.68 ^b^	285.81 ± 21.67 ^b^	285.39 ± 31.31 ^b^
Lysine (mg/kg)	1901.33 ± 358.61 ^b^	1782.28 ± 128.88 ^b^	1576.94 ± 71.21 ^a^	1410.76 ± 141.34 ^a^
Tyrosine (mg/kg)	23.16 ± 4.81 ^a^	46.82 ± 6.71 ^b^	18.82 ± 1.64 ^a^	16.32 ± 2.08 ^a^
Histidine (mg/kg)	1258.02 ± 263.20 ^d^	991.05 ± 386.96 ^c^	586.42 ± 60.92 ^a^	744.16 ± 59.94 ^b^
Total free amino acid content (mg/kg)	16,258.26 ± 628.69 ^c^	14,609.75 ± 626.77 ^b^	11,422.44 ± 484.87 ^a^	10,818.85 ± 466.27 ^a^

C, control treatment, without starter cultures; S2A, treatment with *L. plantarum* S2A; S4B, treatment with *L. pentosus* S4B; OP4, treatment with *L. plantarum* OP4; Different lowercase letters indicate statistically significant differences between groups according to Tukey’s test (*p* < 0.05).

## Data Availability

Data is contained within the article.

## Abbreviations

The following abbreviations are used in this manuscript:

LAB	Lactic Acid Bacteria
TBARS	Thiobarbituric Acid Reactive Substances
CNS	Coagulase-Negative Staphylococci
GRAS	Generally Recognized As Safe
MRS	De Man, Rogosa, Sharpe
PCA	Plate Count Agar
TBX	Tryptone Bile X-Glucuronide
BWP	Buffered Water Peptone
XLD	Xylose Lysine Deoxycholate
MDA	Malondialdehyde
GC-FID	Gas Chromatography With Flame Ionization Detection
UPLC–HRMS	Ultra-High Performance Liquid Chromatography–High-Resolution Mass Spectrometry
ANOVA	Analysis Of Variance
EFSA	European Food Safety Authority
CLA	Conjugated Linoleic Acid
EPA	EicosaPentaenoic Acid
DHA	DocosaHexaenoic Acid
SFA	Saturated Fatty Acid
PUFA	Polyunsaturated Fatty Acid
MUFA	Monounsaturated Fatty Acid
FAA	Free Amino Acid
BA	Biogenic Amine
